# Robust and diverse multidimensional statistical moments in dual-band entomological lidar for improved real-time insect monitoring

**DOI:** 10.1242/jeb.251761

**Published:** 2026-05-28

**Authors:** David Dreyer, Meng Li, Hampus Månefjord, Assoumou Saint-Doria Yamoa, Yatana Adolphe Gbogbo, Lauro Müller, Anna Runemark, Benoit Kouassi Kouakou, Rabbi Boateng, Andrew Atiogbe Huzortey, Jérémie T. Zoueu, Benjamin Anderson, Mikkel Brydegaard

**Affiliations:** ^1^ Department of Physics, Lund University, Sölvegatan 14c, 22363 Lund, Sweden; ^2^ Institut National Polytechnique Félix Houphouët-Boigny de Yamoussoukro, BP 1093 Yamoussoukro, Côte d'Ivoire; ^3^ Department of Biology, Lund University, Sölvegatan 35, 22362 Lund, Sweden; ^4^ Department of Physics, University of San-Pedro, BP 1800 San-Pedro, Côte d'Ivoire; ^5^ Laser and Fibre Optics Centre (LAFOC) at the University of Cape Coast, CC-123-1749 Cape Coast, Ghana; ^6^ Norsk Elektro Optikk, Østensjøveien 34, 0667 Oslo, Norway

**Keywords:** Statistical moments, Entomological lidar, Biodiversity, Remote sensing

## Abstract

As some insect groups are declining at alarming rates, accurate and automated insect monitoring is needed to prioritize habitats for conservation. The dual-band entomological Scheimpflug lidar technique is a promising candidate method for real-time insect monitoring: it allows the detection of thousands of flying insects per day at high temporal and spatial resolutions. The signals contain a plethora of properties which can be assigned to flight heading- and species-specific clues which may improve classification. Here, we introduce a systematic approach to robust dimensionality reduction of entomological lidar range-time intensity matrices (time and range, 2D) of observations, into time dependent vectors (1D) and scalar values (0D) which encode features related to the flight heading and species characteristics. Using this single-night dataset as a case study, we show that dual-band parameters not only confirm expected patterns of average insect melanization but also enable exploration of signal diversity such as insects that display distinct spectral signatures.

## Teaser

**Summary:** Automated monitoring by dual-band Scheimpflug lidar captures thousands of insects, enabling flight-heading and insect-specific analyses for conservation of these vital pollinators, food sources and recyclers.

## INTRODUCTION

In face of the ongoing biodiversity decline (Hallmann et al., 2017), efficient monitoring approaches that can provide data to guide conservation action are crucial. Monitoring of the abundance and diversity of insect communities in different habitats has been established as a measure of the status of a habitat's overall biodiversity (Landmann et al., 2023). Data on insect abundance and diversity are therefore an excellent basis for identifying habitats to target for conservation and validating conservation measures on species composition. However, large-scale and long-term studies (e.g. Basset et al., 2012; Hallmann et al., 2017; Ronquist et al., 2020) targeting the overall diversity of invertebrates in their respective habitats do often exceed the capacities of research groups with limited resources in terms of research budget and workforce.

Entomological Scheimpflug lidars (Brydegaard and Svanberg, 2018; ESLs, ∼50 kg instrument mass) offer cost-efficient, long-term monitoring of insect activity. ESLs feature the ability to record hundreds of thousands of insect observations per day (e.g. Brydegaard et al., 2020) with multi-kilohertz temporal sampling rates and a spatial range resolution of relative accuracy between 3% and 5% of the length of the field of view covered by the beam, which is  unachievable by conventional malaise traps. Although the setup, calibration and operation of an ESL system require technical expertise and experience, ESL systems are conceptually simple, consisting mainly of two main components: a laser transmitter unit and a receiver unit extensively described elsewhere (e.g. Brydegaard et al., 2017; Jansson et al., 2021). In summary, the concept is based on a collimated laser beam (multiband or single band, up to several hundred meters long, ∼75 mm diameter) which is usually pointed at a termination target (a black neoprene surface) for calibration purposes. The beam can be directed horizontally, vertically at the sky, or at any angle in between. When insects fly through the laser beam, light is scattered from their body and wings. This backscattered light is then captured by the receiving telescope and focused onto a sensor. The Scheimpflug configuration (Brydegaard et al., 2017) of the detector allows a large aperture and recording of sharp echoes over several hundred meters at fast pace. We refer to these as insect observations and their range from the instrument can be inferred from pixel number where light is focused on the sensor.

As the linear complementary metal–oxide–semiconductor (CMOS) array can operate at sampling frequencies up to several kilohertz, hundreds of exposures of the same insect can be recorded while it transits the beam. A typical insect observation lasts ∼25 ms and contains hundreds of exposures, featuring an oscillatory signal contribution from the wings and a non-oscillatory contribution from the body (the envelope), allowing us to resolve wing beats and discriminate body to wing sizes for target classification. Diverse signals can form the basis of diversity assessment by unsupervised clustering and machine learning techniques (e.g. Wallace et al., 2023; Rydhmer et al., 2024; Bernenko et al., 2024; Yamoa et al., 2025), which hold the potential for automated genus or even species identification.

One parameter of interest for classification is insect coloration, which provides important cues for species identification but is challenging to capture using lidar. The darkish or brownish body coloration of insects is strongly influenced by the degree of melanin, which governs the production of this pigment only in the cuticle (Popadić and Tsitlakidou, 2021). While generally appearing as shades of brown, the diversity of these colors can be notable as a result of variations in melanin concentration and distribution, even between closely related species (True, 2003). Melanin exhibits stronger absorption at 808 nm than at 980 nm (Stavenga et al., 2015). While the absorbance of insect aero-fauna is generally higher at 808 nm than at 980, the scattering is also stronger at 808 nm, meaning that for a small species, for example, a higher proportion of light at 980 nm may pass through body and be lost on the back side of the insect.

Additionally, specular reflectance from wings can be resonant in either of these two bands. For these reasons, the use of dual-band lidars which are sensitive to melanization, the scattering coefficient relative to body size and the wing membrane thickness relative to the wavelength (Gebru et al., 2017; Li et al., 2020a,b, 2023a,b, 2025; Li, 2024), provides additional information facilitating genus- or species-level identification.

In this work, we introduce a systematic and robust approach to dimensionality reduction of lidar insect observations, based on multidimensional statistical moments providing a reduced set of parameters reflecting instrumentation, flight headings and species-specific features. We applied this approach in exploring information richness of dual entomological lidar signals from a tropical virgin forest using a single-night recording as a case study to present general trends of insect reflectivity due to melanization. We highlight exotic exceptions originating from selected species. These advances suggest that entomological lidar signals are richer in information than previously assumed and that multidimensional parametrization and additional wavelength bands could expand insect classification parameter space to the extent where most coexisting species are differentiated, or certain genera or even species can be pinpointed.

## MATERIALS AND METHODS

### Study site

The Taï National Park was selected for monitoring of insect diversity and abundance as it persists as a largely untouched and vital rainforest. It hosts a 3300 km^2^ patch of pristine tropical, moist broadleaf forest recognized as a UNESCO biosphere reserve. The Taï forest forms part of the Upper Guinean Forest system of Western Africa. Over the last decades, logging and extensive agriculture have threatened the existence of this ‘biodiversity hotspot’ in Côte d'Ivoire (Arcilla et al., 2015). The climate in Taï is seasonally variable (Nicholson and Grist, 2003). Monitoring was performed in January 2023 during the Harmattan (dry) season. The deployment site, located deep within the forest, was strategically selected at a boundary between dense forest and a small clearing at an ecological research station of Taï (Nangui Abrogoua University). The lidar was deployed at a distinct forest edge providing an unobstructed lidar monitoring path. Monitoring commenced at approximately 20:00 h local time on 7 January 2023, well within astronomical darkness at the recording site (sunset: 18:20 h local time). The laser beam was directed to penetrate the canopy and point towards the night sky, to detect insects flying beyond and above the canopy.

### Configuration of the lidar system

The dual-band ESL system operates at wavelengths of 808 and 980 nm. The ESL system was designed according to the Scheimpflug principle, to allow for high-resolution detection over extended distances. This was achieved by aligning the transmitter and receiver telescopes, which were mounted on a tripod (EQ8, SkyWatcher), and with a baseline separation of 814 mm. The transmitter consisted of a 75 mm diameter, 300 mm focal length telescope, with an attached module containing two 3 W TE-polarized laser diodes emitting at 808 and 980 nm. The sources were superimposed by a dichroic mirror. The receiver module comprised a Newton reflector telescope (Teleskop Service, Vaterstetten, Germany) with a 150 mm diameter and 600 mm focal length. A linear CMOS camera (OctoPlus, Teledyne e2v, Waterloo, ON, Canada), with 2048 pixels (10×200 μm^2^ each), was installed in the focal port of the receiver. A 3D printed camera holder featured an angle of 37 deg between the camera and the optical axis to adhere to the Scheimpflug condition.

The lidar system, mounted 2 m above ground level at the forest boundary and tilted upward at a 10 deg angle, projected its beam upward from 2 m above ground level across the top of the rainforest canopy (25 m tall). The system's probe volume (the overlap of its beam and field of view) extended from 20 m to infinity. The camera was operated at a 10 kHz line rate. Because of multiplexing with three time slots (808 nm, 980 nm and background; see Zhao et al., 2018), the effective sampling frequency per band was 3.33 kHz, and the exposure time was 80 μs. The system generated 3 s raw data files, each containing 30,000 scan lines with a file size of 120 MB. Backscattered light intensity was recorded with 12-bit resolution (0 to 4095 counts). A custom LabVIEW script continuously logged the lidar raw data binary files for subsequent analysis and interpretation. Over the duration of the measurement campaign, a total of 1.1 TB of raw data was acquired. After cropping out insect observations, the dataset was reduced to approximately 1% of its original size.

The recorded files were loaded into MATLAB and demultiplexed into three 2D matrices *I*_808_(*p*,*t*), *I*_980_(*p*,*t*) and *I*_bgr_(*p*,*t*). These matrices represent the backscattered intensity at each pixel, *p*, and exposure, *t*. After background subtraction, the data were subsequently cropped (Malmqvist et al., 2016; Li, 2024). This process involves creating a Boolean range-time mask where backscatter in a pixel exceeds a threshold. This threshold is defined as the temporal median plus five times the interquartile range of that same pixel within a 3 s file. Islands of connected elements in time and space within the mask are identified, cropped out and hereafter referred to as observations (see Fig. S1). As the distance and reflectance of the black neoprene target are known, the intensity signal of each insect observation can be calibrated to the optical cross-section, σ, in mm^2^ for each observation (Brydegaard et al., 2021).

### Primary statistical moments (from 2D to 1D)

In this section, the systematic reduction of 2D range-time intensity matrices into five 1D time-dependent vectors by applying primary statistical moments is described. These vectors can in turn be reduced to scalar values (0D) which describe distinct properties of insects and their movement. This complete, two-step process, from 2D matrix to 1D vector (primary moments) and finally to 0D scalar (secondary moments), is systematically summarized in Table 1, where variable names denote *p* for pixels, *t* for time (ms), σ for cross-section (mm^2^), *r* for range (m) and δ for apparent size (mm). The superscript denotes the primary moment in the pixel domain and the subscript denotes the secondary moment in the time domain. For example, σ^1^_0_ describes the mean cross-section (body and wings) of an observation over time whereas σ^2^_0_ describes the cross-section variation (wing cross-section).

**Table 1.** A systematic overview of the statistical moments and their meaning in entomological lidar data

Primary statistical moments	Time-dependent vectors	Secondary statistical moments				
		0th (sum)	1st (mean)	2nd (spread)	3rd (skewness)	4th (kurtosis)
0th (sum)	Σ*p*_(_*_t_*_)_ *→* σ_(_*_t_*_)_	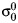	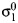	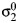	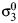	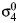
			Body cross-section	Wing cross-section	Glossiness	
		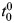	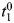	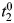	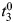	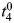
			Absolute time	Transit time	Signal decrease	Beam shape
1st (mean)	*p*_CoM(_*_t_*_)_ *→ r*_(_*_t_*_)_	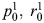	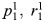	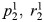	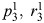	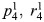
			Mean range	Displacement	Curvilinearity	Erraticness
		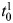	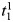	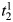	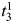	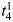
					Distance/altitude decrease	
2nd (spread)	*p*_spread(_*_t_*_)_ *→* δ_(_*_t_*_)_	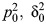	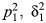	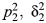	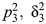	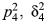
			Apparent size	Wing size		
		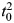	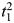	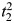	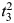	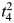
					Size decrease	
3rd (skewness)	*p*_skew(*t*)_	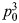	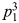	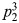	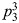	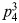
			Dorsal/ventral	Wing amplitude		
		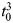	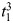	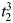	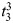	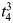
4th (kurtosis)	*p*_kurt(*t*)_	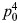	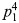	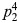	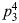	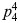
			Optical focus			
		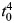	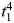	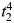	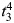	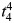

The primary statistical moments (sum, mean, spread, skewness and kurtosis) were calculated in the pixel domain from the time-dependent vectors listed in the second column. The secondary statistical moments were applied in the time domain to those same vectors. The temporal vectors can in turn be described by their statistics on both their magnitude distribution and the temporal shape. The physical interpretation of each parameter is given by its variable name, where *p* is pixels, *t* is time (ms), σ is cross-section (mm^2^), *r* is range (m) and δ is apparent size (mm). The superscript denotes the order of the primary moment, and the subscript denotes the order of the secondary moment. Parameters are color coded by their primary relevance: species specific (green), trajectory related (blue) and instrument related (red). All other parameters were not explored further in this study.

The retrieved backscattered intensity for each observation is treated individually in each band and hereafter referred to as *I*_(*p*,*t*)_. It forms a *P×T* matrix of 12-bit light-intensity counts, where *P*∈{*p*_near_… *p*_far_} are the range pixels and *T*∈{*t*_first_… *t*_last_} are the exposures of the observation mask. Using statistical moments, this matrix is reduced to five time-dependent vectors – lidar optical cross-section, center of mass, spread, skewness and kurtosis.

Here, *I*_(*p*,*t*)_ is the signal strength of pixel *p* during exposure *t* (see Fig. S2). The cross-section is determined from the 0th moment, calculated by summing all range pixels per exposure and denoted as Σ*p*_(_*_t_*_)_ (Eqn 1). The subsequent moments calculated are the 1st moment: the absolute pixel center-of-mass *p*_CoM(*t*)_ (Eqn 2); and the 2nd moment: the differential pixel spread denoted *p*_spread(*t*)_ (Eqn 3):
(1)

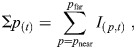

(2)

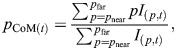

(3)

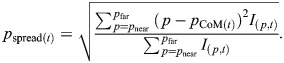


**Fig. 1.**
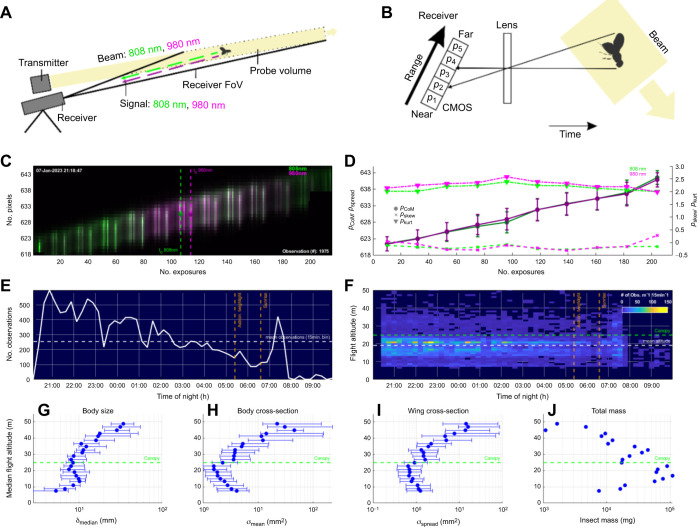
**Dual band entomological lidar and example outputs.** (A) Schematic diagram of the dual-band lidar setup. FoV, field of view. (B) Example of an insect crossing the lidar beam, with backscattered light registered on different complementary metal–oxide–semiconductor (CMOS)-sensor pixels (*p*). (C) False-color 2D map of a single insect observation (green, 808 nm; magenta, 980 nm), showing the signal strength (12-bit resolution). Dashed lines mark the calculated center of mass (*t*_0_, *t*_1_^0^ in Table 1). (D) Statistical moments (shown every 20th exposure). Filled circles depict the pixel center of mass (*p*_CoM_), and vertical error bars indicate the spread (*p*_spread_). Crosses and triangles represent skewness (*p*_skew_) and kurtosis (*p*_kurt_). (E) Number of observations over time (15 min bins). (F) 2D histogram of flight altitude versus time of night. (G–J) Flight altitude (2 m bins) plotted against median apparent body size δ_median_ (G), mean body cross-section σ_mean_ (H), mean wing cross-section σ_spread_ (I) and total insect mass per altitude layer (J). Bars in G–I show the 25th–75th percentiles (interquartile range, IQR).

Representative examples of these quantities are plotted in Fig. 1. To reduce the dimensionality of the signal distributed over a respective range of pixels, its skewness and kurtosis were calculated using p_CoM(*t*)_ and *p*_spread(*t*)_:
(4)

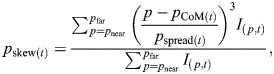

(5)

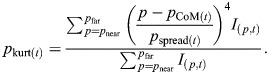


### Calibration from pixel to metric units

**Fig. 2.**
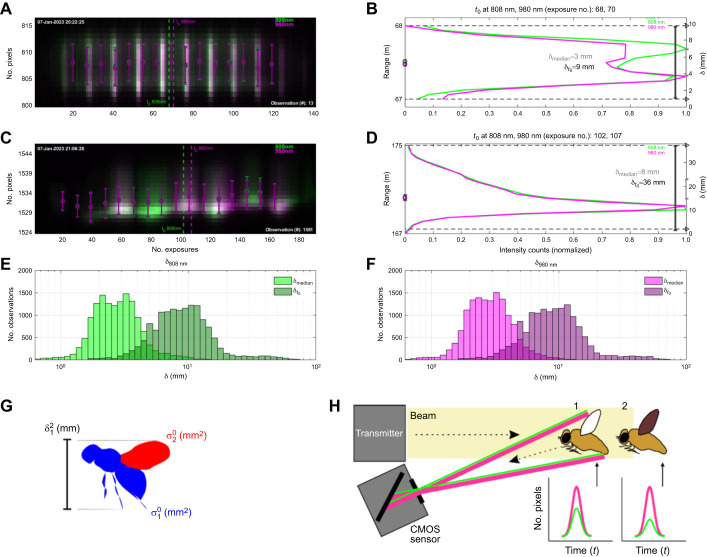
**Estimating apparent target size from dual-band lidar observations.** (A,C) Time-range maps of the dual-band signal (green, 808 nm; magenta, 980 nm). Dashed lines show exposure numbers closest to the calculated center of mass (*t*_0_). (B,D) Apparent size and range of the insects in A and C. Open circles on the left *y*-axis indicate the mean range (*r*_1_^1^ in Table 1). Apparent size (δ_1_^2^ in Table 1) is calculated using two methods (see Materials and Methods). Method 1 uses the intensity spread over pixels, while method 2 thresholds pixels at *t*_0_ and projects the apparent size on a precalculated scale. (E,F) Histograms of apparent sizes for both methods (E, 808 nm; F, 980 nm). (G) Schematic differences between apparent size (δ_1_^2^) and cross-sections (σ_1_^0^, σ_2_^0^). (H) Illustration of an insect crossing the beam with distinct scattering at 808 nm and 980 nm.

Each absolute pixel number in the center-of-mass vector, *p*_CoM(*t*)_, corresponds to a specific range from the lidar, denoted as range *r*_(*t*)_ (in m). This range is calculated according to Eqn 6:
(6)

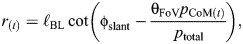
where *p*_total_ denotes the total number of pixels on the linear array (2048 pixels); *ℓ*_BL_=814 mm is the length of the baseline; φ_slant_=1.51 deg is the angle between the optical axis of the beam expander and receiver (the slant angle was determined by an echo from a fixed point at a known range); and θ_FoV_=1.56 deg is the field of view, obtained from the ratio between the tilted array length and the receiver focal length. The temporal mean of range vector, *r*_mean_, is highlighted as an example in Fig. 2. Further details on the lidar system design can be found in Kouakou et al. (2020) and Brydegaard et al. (2021). The differential pixel spread, *p*_spread(*t*)_, represents an apparent size, δ_(*t*)_ (in mm), which is calculated for each time *t* as:
(7)

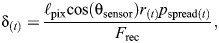
where *ℓ*_pix_=10 μm is the pixel pitch, θ_sensor_=37 deg is the sensor tilt and *F*_rec_=600 mm is focal length of the receiving telescope.

Two different approaches can be employed to calculate this apparent size (labeled δ_1_^2^ in Table 1): method 1 (conventional) uses the spread p_1_^2^ over the respective pixels across all exposures (depicted as the distance between the light gray error bars in Fig. 2B,D), while method 2 thresholds only the pixels containing the signal at *t*_0_ and projects the resulting size on a previously calculated scale (dark gray error bars in Fig. 2B,D). Although method 1 tends to give a more conservative estimate, it can underestimate individual observations; in contrast, method 2 may yield larger apparent sizes but can also be more sensitive to noise in the thresholding step (see Fig. S6). Based on these characteristics, we recommend method 1 as the default for general monitoring and low-signal to noise ratio (SNR) datasets, while method 2 is preferred for high-SNR observations where a precise determination of the insect's dimensions at the beam center is achievable.

The echo magnitude, Σ*p*_(*t*)_, corresponds to an equivalent white target with the backscatter cross section, σ_(*t*)_ (in mm^2^), which is calculated as:
(8)

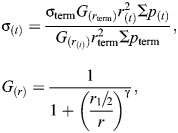
where σ_term_ is the cross-section of the termination echo, Σ*p*_term_, on a neoprene target with known reflectance of 2% across near-infrared. The termination target was placed at *r*_term_=80 m as this represented the furthest ground distance available providing a clear, accessible transect for calibration during the campaign. Further, *r*_term_=80 m is the termination to the termination target, *G*(*r*) is the lidar range-dependent gain, which follows a sigmoidal functional form, previously established (Brydegaard et al., 2021) to describe the geometric overlap in Scheimpflug lidar systems. The parameters *r*_½_=56 m, the range of half beam-field-of-view overlap, and γ=1.4, the slope of the gain function, were determined by fitting the analytical function with homogeneous atmospheric lidar signals using MATLAB cftool. We acknowledge that this calibration assumes a stable gain beyond the termination; however, the Scheimpflug geometry introduces a range-dependent sensitivity bias. At close range, the triangulation angle maps small beam segments to many pixels, creating high-resolution sampling volumes. Conversely, at greater distances, longer beam segments are compressed into fewer pixels, increasing the sampling volume and favoring the detection of larger or more specular targets.

### Secondary statistical moments (from 1D to 0D)

The five time-dependent vectors from the pixel domain moments in Eqns 1–5 (and calibrated parameters thereof), can be further reduced to scalars by applying statistical moments to the quantities or in time. An overview is given in Table 1.

**Fig. 3.**
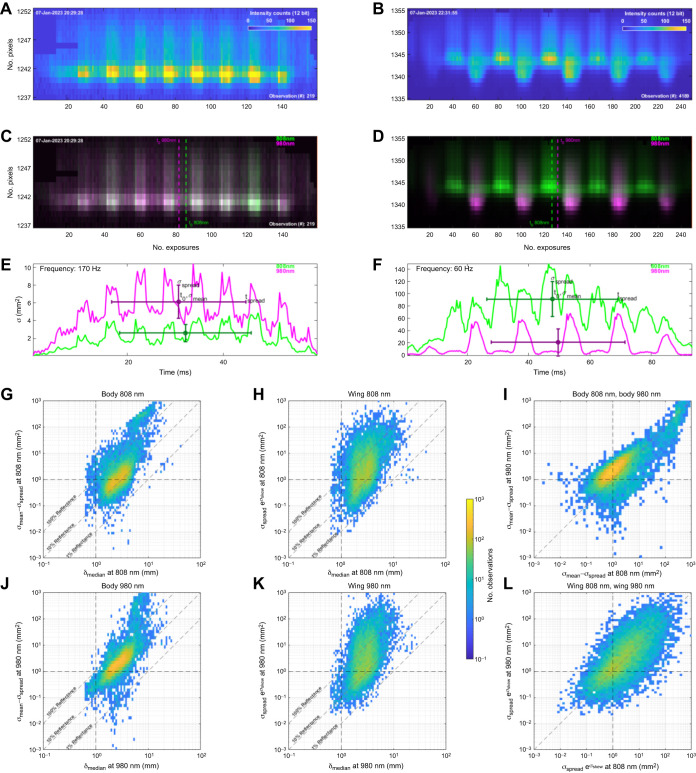
**Comparison of insect cross-sections (body and wings) in both signal bands (808 and 980 nm).** (A,B) Time-range maps of two insect recordings. (C,D) Dual-band time-range maps shown in false colors (green, 808 nm; magenta, 980 nm). Dashed lines mark the exposure closest to the signal's center of mass (*t*_0_). (E,F) Cross-sections over time; filled circles represent the mean cross-section (σ_mean_), with vertical error bars indicating the spread (σ_spread_). (G,J) 2D histograms of body cross-sections (σ_mean_−σ_spread_) plotted against apparent size (δ_median_). (H,K) 2D histograms of wing cross-sections (σ_spread_). (I,L) Direct comparisons of body cross-sections (I) and wing cross-sections (L) between the two bands. Note: in H, K and L, skewness σ_skew_ is expressed as an exponential e^σskew^ to ensure a strictly positive scale and to improve the visual distribution and separation of the signal clusters.

To determine the proportion of the wings (σ_2_^0^ in Table 1) in the total cross-section of an insect, the second statistical moment, the spread (σ_spread_, see vertical bars in Fig. 3E,F) is used. By calculating the mean or median of δ_(*t*)_, δ_mean_ or δ_median_ in mm is obtained, respectively. We recommend δ_median_ as a more robust metric for practical adoption, as it is less sensitive to the non-Gaussian intensity distributions and specular outliers commonly produced during wing flashes.

**Fig. 4.**
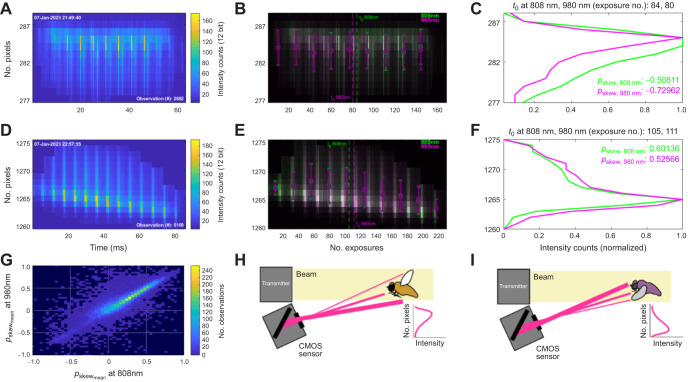
**A comparison of signal skewness in the 808 and 980 nm bands.** (A,D) Time-range maps of two insect recordings. (B,E) Dual-band time-range maps shown in false colors (green, 808 nm; magenta, 980 nm). Circles mark the center of mass of the intensity distribution; vertical error bars show the spread. (C,F) Normalized intensity distributions of B and E, illustrating negative skew (C) and positive skew (F). (G) 2D histogram comparing skewness in the 808 nm versus 980 nm bands. (H,I) Schematic diagram showing how signal skewness relates to wing reflectivity: negative skew often indicates reflection during the downstroke (H), positive skew during the upstroke (I).

The mean skewness (*p*_skew mean_, *p*_1_^3^ in Table 1) and mean kurtosis (*p*_kurt mean_, *p*_1_^4^ in Table 1) of the intensity distribution are statistical features that describe certain insect-intrinsic properties. For example, a negatively skewed distribution of the insect signal intensity means that the wings reflect light during the downstroke of the wingbeat cycle (refer to Fig. 4, and in particular Fig. 4C,H, which show example signals with negative skewness).

The kurtosis of the intensity distribution can be used to make estimations about the reflectiveness of the entire insect (see examples shown in Fig. S8). The body of an insect usually shows a higher degree of diffuse reflection compared with its wings or legs. This leads to a high kurtosis of the overall signal's intensity distribution if the thorax and abdomen of an insect are small compared with the less melanized surface (see Fig. S8C,H).

To identify the most representative exposure number of the insect's transit, which is weighted by the signal strength or when the insect was most prominently detected by the lidar system, *t*_0_ (*t*_1_^0^ in Table 1; see dashed green and magenta lines in Fig. 1C), the center of mass of *t* is calculated. Eqns 9 and 10 give the mean of the pixel domain and time domain, respectively:
(9)

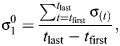



(10)

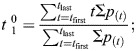
Eqns 11 and 12 give the spread of the pixel domain and time domain, respectively:
(11)

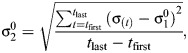

(12)

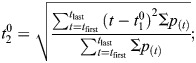
Eqns 13 and 14 give the skewness of the pixel domain and time domain, respectively:
(13)

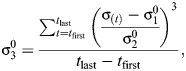

(14)

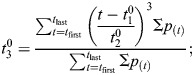
and Eqns 15 and 16 give the kurtosis of the pixel domain and time domain, respectively:
(15)

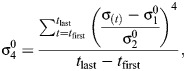


(16)

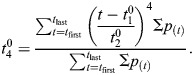


In addition to the absolute time of each observation, the transit time of an insect (*t*_spread_, *t*_2_^0^ in ms in Table 1) can be determined by calculating the spread of the signal intensity over the exposures of a given insect observation and dividing the result by the sampling rate (*f*_s_). *t*_2_^0^ quantifies the spread of the signal around *t*_1_^0^, in the time domain. It gives the duration of the event (the insect crossing the lidar beam). A smaller *t*_2_^0^ indicates that the signal is tightly clustered around *t*_1_^0^, meaning the insect crossed the beam quickly and at a consistent speed, while a larger *t*_2_^0^ suggests a slower crossing or crossing at varying speeds. The skewness (*t*_3_^0^) and kurtosis (*t*_4_^0^) of the signal in the time domain can be used to determine whether the insect of a respective observation was flying away from or towards the receiver and to describe the ‘envelope shape’ (see Fig. S3). It is important to note that these time-domain moments represent the convolution of the insect's transit with the lidar's spatial instrument function. For a symmetric Gaussian beam, the theoretical baseline for skewness *t*_3_^0^ is 0 and that for kurtosis *t*_4_^0^ is 3. We interpret deviations from these baselines as biological indicators, such as radial trajectories (approaching or receding) or erratic flight behavior, that cause the signal envelope to depart from the instrument's expected symmetric profile.

As the secondary statistical moments described here are standard repetitive arithmetic operations, which are each applied to the respective time-dependent vectors (see Table 1), the individual arithmetic steps are exemplified only for the pixel and time domains of the cross-sections (see Eqns 9–16). A detailed description of the other metrics can be found in the Results and Discussion, and in Fig. S3.

## RESULTS AND DISCUSSION

### General activity patterns

During the night of 7–8 January 2023, the system recorded over 14,310 observations of insects at various heights; examples of observations are shown in Fig. 1. The rate of observations peaked at 21:00 h and began to gradually decline from around 23:00 h onwards until it fell below the mean observation rate (255 observations per 15 min bin) at around 04:00 h. This pattern was interrupted by a narrow peak after sunrise between 07:00 h and 08:00 h (Fig. 1E). The mean (±s.d.) flight altitude of the detected insects was 19.2±8.7 m, as highlighted in Fig. 1F, while the highest detected observation was at an altitude of about 405 m. The peak flight activity took place right under the canopy (∼25 m) at an altitude between 20 and 25 m during the first half of the night. This altitude range is further explored in Fig. 1G–I, showing the detection of apparent size, body cross-section and wing cross-section by height; Fig. 1J indicates the highest dry mass concentration was also found here.

### Body-specific optical moments

#### Apparent size (δ)

Our data indicate a positive correlation between insect size and flight altitude (Fig. 1F), suggesting that larger insects tend to fly at higher altitudes (but see Discussion). Dual-band lidar apparent size extraction is exemplified in Fig. 2A–D, showing how the intensity distributions for each wavelength are captured and processed. Notably, dual-band lidar calculations revealed two approaches for estimating apparent size (δ_1_^2^ in Table 1). Method 1 delivered a more conservative impression but possibly underestimated individual observations; method 2 often yielded larger apparent sizes. Across the examples shown in Fig. 2B,D, the mean ranges (*r*_1_^1^) of these observations were between 67 and 68 m for one insect and around 170 m for another. Differences in signal strength between the two wavelengths may result from spectral properties of the insects, including body melanization and wing interference effects arising from variation in wing thickness. This can cause pronounced differences in the signal ratio, even for closely related species. Fig. 2E,F presents the distribution of observations across different apparent sizes for the 808 and 980 nm bands, respectively. The mean (±s.d.) apparent size of our observations was 3.12±3.7 mm for the 808 nm signal-band and 3.19±8.2 mm for the 980 nm signal-band. Fig. 2G conceptually distinguishes apparent size (δ) from wing/body cross-sections (σ), and Fig. 2H schematically represents wavelength-dependent scattering, as due to the spectral characteristics of the given insect or its body parts (in this scheme, the wings). The ratio of the signal strength of both bands can differ profoundly, even if closely related species are registered. Insect posture during flight, including abdomen pitch and wing phase, depends on the respective species and flight conditions (e.g. wind speed) and can affect the calculated apparent size. Apparent size, or δ, should therefore be understood as a posture-dependent proxy rather than a definitive measure of size. As a result, the apparent size of a species may vary across different flight conditions or between an individual in flight and in another behavioral context (e.g. walking or stationary). The Scheimpflug lidar observes scattered light at different angles. Absolute angles are interpreted as a range via triangulation, while differential angles – such as spread – represent an angular spread that is multiplied by range to infer metric size.

#### Body and wing cross-sections (σ)

Based on the sum of the calculated body cross-sections (∼0.23 m^2^), the total mass of detected insects was estimated to be approximately 0.58 kg using a formula and coefficients suggested by Genoud et al. (2023). Examples of two insect observations are shown in Fig. 3. The filled circles in Fig. 3E,F represent the mean cross-section (σ_mean_) of all exposures at *t*_0_ (*t*_1_^0^ in Table 1), while the vertical error bars depict the corresponding spread (σ_spread_, σ_2_^0^ in Table 1) within the measured time window (*t*_spread_, *t*_2_^0^ in Table 1). This spread σ_2_^0^ primarily reflects the wing signal; however, it is not an exclusive measurement of wing size. Factors such as specular flashes (wavelength dependent), the specific phase within a wingbeat and the insect's posture can all influence the resulting spread. This σ_spread_ primarily captures the wing signal. The cross-sections of the 980 nm are larger than the cross-sections of the 808 nm signal band in most recordings as shown in Fig. 3. Specifically, in about 80% of observations, the 808 nm signal is weaker than the 980 nm signal (see Fig. 3E), but in ∼20% of the body cross-section recordings (σ_1_^0^ in Table 1; Fig. S2) the 808 nm band is higher. Similarly, ∼30% of the wing cross-sections (σ_2_^0^ in Table 1) also show a stronger 808 nm response (Fig. 3I). Scatterplots of median offset (δ_median_) against spread (σ_spread_) in Fig. 3G,H,J,K show that wing and body returns form separate clusters, indicating different scattering characteristics at each wavelength. The opposite is the case for only ∼20% of observations for the body cross-sections (σ_1_^0^ in Table 1; see Fig. S2) and ∼30% for the wing cross-sections (σ_2_^0^ in Table 1; see Fig. 3I). For many insect bodies (Fig. 3G,J), the signal at 980 nm was observed to be nearly twice as strong as the signal at 808 nm, probably due to weaker melanin absorption and consequently higher diffuse reflectance at 980 nm. The recorded mean (±s.d.) body cross-sections were 16.3±70.2 mm^2^ at 808 nm and 18.2±89.7 mm^2^ at 980 nm. The corresponding mean (±s.d.) wing cross-sections were 3.9±14.3 mm^2^ and 6.12±27.1 mm^2^, respectively.

### Statistical moments

#### Skewness (*p*_skew_)

Depending on the insect species, the ventral or dorsal side of the wings (Fig. 4A,B) displays a higher degree of reflection (Fig. 4D,E). This depends on whether the specific exposure measured the animals' signal during the downstroke (Fig. 4H) or the upstroke (Fig. 4I) of the wingbeat cycle. As the signal from the body of an insect is usually stronger than the signal from its wings, the distribution of the signal's intensity usually shows some degree of skewness in both signal bands, which can be negative or positive (see also Fig. S1). A positive or negative mean skewness of the signal in the pixel dimension, *p*_skew_ _mean_ (*p*_1_^3^ in Table 1) provides insight into the distribution of the signal along the dorsoventral axis of the insect. Note that Fig. 4C,F illustrates examples of negative and positive skewness, respectively, and Fig. 4G compares skewness values across all observations. The configuration of transmitter and receiver was ‘upside-down’ along the horizontal axis while the shown dataset was recorded. Therefore, the depicted observations report a signal from the ventral side of the wing (see Fig. 4A,B,H) and from the dorsal side of the wing (see Fig. 4D,E,I).

#### Kurtosis (*p*_kurt_)

Some insect species have a larger reflective body surface relative to their overall mass than others. Two distinct insect observations (illustrated in Fig. S8) exhibit different kurtosis values in their reflection signals. Crane flies, for instance, spread out their legs while in flight, which makes their cross-section much larger relative to the actual diameter of the thorax and abdomen (Fig. S8I, right). Moths, in contrast, tuck their legs close to their abdomen while in flight, creating a smaller cross-section relative to the mass of the insect. This leads to varying kurtosis values of the mean intensity distribution of the signal (Fig. S8C,F) in the pixel domain, *p*_kurt_ _mean_ (*p*_1_^4^ in Table 1), encoding a varying ‘signal compactness’. While the 980 nm band in Fig. S8C exhibits a more compressed, low-kurtosis distribution, the same band appears high in kurtosis in Fig. S8F; the 808 nm signal shows the opposite configuration. Because the body of an insect typically has stronger diffuse reflection than its wings or legs, overall signal kurtosis can be high, if the thorax and abdomen of an insect are small if compared with the overall less melanized surface (as in the crane fly in Fig. S8H) or if the thorax and abdomen are big and highly melanized (as in the beetle in Fig. S8I). This variability also appears in the 2D histogram of kurtosis values (Fig. S8G) and the composition diagram (Fig. S8H), underscoring how the two bands (808 nm and 980 nm) can yield markedly different kurtosis measurements.

### Trajectory-related statistical moments

**Fig. 5.**
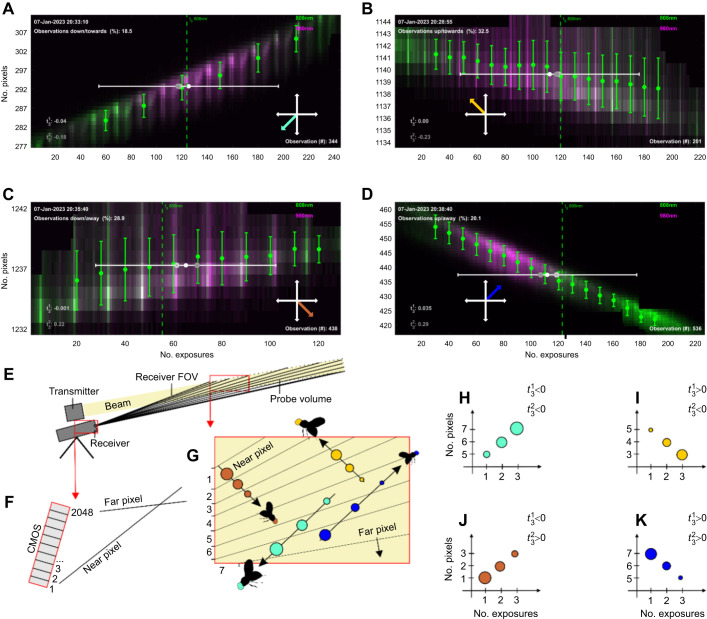
**Trajectory-related statistical moments.** (A–D) Time-range maps of four insect observations (808 nm signal shown). White circles show the mean *p*_CoM(*t*)_ *p*_1_^1^ with a horizontal bar indicating its time-domain spread *t*_2_^1^. Vertical green bars depict *p*_spread(*t*)_. Light-gray and dark-gray circles on the horizontal bar represent skewness in *p*_CoM(*t*)_ *t*_3_^1^ and *p*_spread(*t*)_ *t*_3_^2^, respectively. (E,F) Schematic diagrams of the CMOS sensor's partial volumes: near observations appear on ‘near pixels’, far observations on ‘far pixels’. (G) Circle diameter encodes the angular size *p*_spread(*t*)_; circles grow if an insect flies toward the receiver and shrink if it flies away. (H–K) Illustrations of negative versus positive skewness of *p*_CoM(*t*)_ (flying down versus up) and *p*_spread(*t*)_ (toward versus away). Arrow colors in A–D match the circle colors in G and dot colors in H–K.

We propose that certain statistical moments could be used to determine the trajectory which an insect followed while it was flying through the laser beam, under the assumption that the beam profile is homogeneous and the beam and camera field of view (FoV) are perfectly aligned. Fig. 5 illustrates how the skewness values *t*_3_^1^ and *t*_3_^2^ could potentially be used in combination to deduce whether an insect was flying up or down as well as towards or away from the receiver. During these recordings, the receiver was installed below the beam (‘viewing upwards’), effectively dividing its FoV into partial volumes (Fig. 5E,F). Observations closer to the receiver focus on higher pixels (‘near pixels’), while those farther away focus on lower pixels (‘far pixels’). Our current interpretation is that when an insect moves toward the receiver, its angular size *p*_spread(_*_t_*_)_ increases; it decreases if the insect flies away (Fig. 5G). Based on this geometric model, a negative skewness of *p*_CoM(_*_t_*_)_, *t*_3_^1^<0 would indicate the insect was flying downward (Fig. 5H,J), while a positive skewness *t*_3_^1^>0 would indicate the insect was flying upward (Fig. 5I,K). Simultaneously, a negative skewness of *p*_spread(_*_t_*_)_, *t*_3_^2^<0 would signify movement toward the receiver, and a positive skewness *t*_3_^2^>0 implies flight away. While these moments provide a robust mathematical description of transit asymmetry, we acknowledge that in the absence of external ground-truth validation (e.g. calibrating with targets of known optical projection across controlled trajectories), these classifications should be viewed as theoretical trajectory proxies rather than definitive measurements. Analysis of these statistical moments describes both the vertical and longitudinal components of insect flight trajectories. About 52% of insects exhibited ascending trajectories and 48% descending trajectories.

The present work extends dual-band entomological lidar from the Ecuadorian cloud forest (Santos et al., 2023) into a West African canopy stand, and couples it with a multidimensional moment framework for every transit. The dual-band lidar configuration provides complementary information from two wavelength bands, enabling improved species discrimination. Single-band lidar typically provides insufficient detail for precise species identification, as metrics such as wing beat frequency or apparent size alone are rarely species specific. Two closely related insect species, or males and females of the same species, may appear nearly identical with respect to wing beat frequency, apparent size and cross-section of wings and body. However, sex- and species-specific differences in pigmentation are common in insects (True, 2003). Differences in the ratio of signals from 808 nm and 980 nm bands might thus help to differentiate species or sexes in sexually dimorphic species which only differ in pigmentation. As the 808 nm band is closer to the absorption band of melanin, we generally expect a lower degree of reflectance at 808 nm compared with the 980 nm signal for diffuse surfaces. While this holds for insect bodies and diffuse wings, the ratio between wavelength bands for clear wing membranes can invert contrasts depending on the specific membrane thickness. These thickness resonances mean that two near-infrared bands alone are insufficient to determine membrane thickness without additional experimental controls such as more spectral bands.

The canopy layer is known to feature a lower insect species diversity and abundance compared with lower strata (de Souza Amorim et al., 2022). However, the species compositions (de Souza Amorim et al., 2022) and their diel activity patterns (Månefjord et al., 2025) also differ from those at lower strata. We found a slight overrepresentation of larger insects observed at higher altitudes (see Fig. 1G–I), potentially partly explained by the fact that larger insects exhibit stronger backscatter, implying that their probability of detection is inherently higher. At least in the Amazon region, upper canopy layers show a high abundance of herbivorous moths and butterflies, which on the whole have larger wingspans compared with other insect groups (de Souza Amorim et al., 2022). Moreover, a high abundance of dragonflies flying above the canopy was observed by members of the crew in the evening (Fig. S7).

Diverse vegetation, including broadleaf trees and epiphytic plants, was abundant at the measurement site (Fig. S7A,B). These plant groups are known to attract insect visitors, including moths, nocturnal bees, crickets and flies (Ray and Gillett-Kaufman, 2022; Balducci et al., 2020). Thus, the high insect density detected beneath the canopy probably reflects activity associated with this complex habitat structure, highlighting the potential for future lidar studies to map insect foraging on, for example epiphytic plants.

As the 808 nm band is closer to the absorption band of melanin, we did expect a lower degree of reflectance at 808 nm compared with the 980 nm signal. This was the case in most but not all observations, which is interesting and will be a matter of further investigation. For example, we documented distinct differences between the intensity profiles of the two wavelength bands in signal strength and shape, as illustrated in the example in Fig. 3E,F. The wingbeat frequency, width of the signal peaks (Fig. 3F), diffuse reflection characteristics (Li et al., 2022; typical for Lepidoptera) and detection timing indicate that this observation represents a moth. In this case, the 808 nm signal (green) is considerably stronger than the 980 nm signal (magenta), contrary to previous measurements of lepidopteran wings (Bosi et al., 2008). The 808 nm band (Fig. 3F) exhibits approximately twice as many peaks as the 980 nm band. Furthermore, the missing peaks in the 980 nm band (Fig. 3F) suggest that the ventral and dorsal sides of the wings have different reflective characteristics.

In Scheimpflug lidar and triangulation systems, range uncertainty is primarily tangential and can be approximated by a quadratic function (Mei and Brydegaard, 2015). For converging beams, the range uncertainty can be approximated as linear and is of the order of 3–5% for the present system (Brydegaard et al., 2017; Malmqvist et al., 2018a).

The accuracy of cross-section estimation is relatively low and, at best, reliable only to the order of magnitude. This metric has previously been used to distinguish insects from vertebrates (Jansson et al., 2023; Malmqvist et al., 2018b; Jansson et al., 2020). In addition to the observational aspects already discussed, insects may traverse the probe volume peripherally, resulting in weaker recorded signals.

The spatial resolution is higher at close range. The resolution is constrained by the point spread function and therefore by the degree of focus. If an insect deviates from the Scheimpflug focal plane of the pixel footprint, the focus deteriorates; in Newtonian telescope configurations, defocusing can lead to bimodal echoes and a reduction in range kurtosis.

The motivation for introducing the beam–FoV overlap function is threefold. First, its range must extend from 0% (no overlap directly in front of the lidar) to 100% (complete overlap at large distances). Second, it should represent a physical quantity and therefore be expressible as an analytical, differentiable function. Third, the function is sigmoidal, increasing monotonically with range, analogous to previously proposed lidar form factors for time-of-flight lidar systems (Li et al., 2023b) and high dynamic range (HDR) lidar (Brydegaard et al., 2021). Calibration plots of the air signals can be found in Fig. S9.

Metrics such as mean body cross-section (σ_mean_, σ_1_^0^ in Table 1) and associated kurtosis in the time domain (*t*_4_^0^ in Table 1; ‘envelope shape’, Fig. S3) are intuitive, whereas other moments encode less intuitive information (Fig. S4). Moments *p*_2_^1^, *p*_3_^1^, *p*_4_^1^, *t*_3_^0^, *t*_3_^1^, *t*_3_^2^ and *t*_4_^1^ (Table 1) bear the potential to describe insect trajectories during beam crossing. The moment *p*_2_^1^ (or *r*_2_^1^ for range) represents the spread of the intensity center of mass, *p*_CoM(*t*)_, across exposures in the pixel domain, distinct from the mean intensity spread (*p*_spread_), which is used to derive δ_mean_ or δ_median_. A low *p*_2_^1^ value might indicate an insect flying straight and perpendicular to the beam, while a high value would indicate lateral displacement (‘zig-zagging’; e.g. Voss and Zeil, 1998) or vertical movement (Fig. S3).

If the suggested variables are taken to explain directionality in space, the skewness of the center of mass, *p*_CoM(*t*)_, and intensity spread, *p*_spread(*t*)_, calculated over the entire observation (*t*_3_^1^ and *t*_3_^2^ in Table 1), would be interpreted as encoding vertical movement (ascending or descending, *t*_3_^1^) and radial movement (approaching or receding, *t*_3_^2^) of the insect (Fig. 5A–D). Additionally, skewness of the sum cross-section in the time domain (*t*_3_^0^ in Table 1; Fig. S3) would quantify changes in the insect's angular size, potentially providing another metric for radial movement. Skewness (*p*_3_^1^) and kurtosis (*p*_4_^1^) of *p*_CoM(*t*)_ in the pixel domain (Table 1; Fig. S3) may help to identify changes in the insect's trajectory while crossing the lidar beam. However, these interpretations would require experimental verification to confirm that these variables reliably capture directional movement. Related approaches describing vertical movements of flying insects using entomological lidar and radar have been reported previously (e.g. Drake and Reynolds, 2012; Drake and Wang, 2018).

The obvious limitations of the method include the as yet low taxonomic resolution which is generally restricted to wing-beat frequencies and size-based groups. Detection is biased toward larger insects and lower altitudes, as signal strength decreases rapidly with range and atmospheric attenuation, limiting sensitivity to larger insects at greater distances. Measurements are further influenced by insect orientation and flight behavior, introducing sampling biases, while adverse atmospheric conditions such as fog, rain or high aerosol loads can degrade data quality. Although lidar provides detailed information on insect position, movement and optical characteristics, complementary methods may provide important biological context regarding specific taxonomic identity, necessitating complementary methods for ecological interpretation.

Insect traps offer high specificity as a result of, for example, genetic identification and the ability to perform dietary and isotopic analyses. However, they require expensive and tedious catch analysis.

Nevertheless, this study demonstrates that the dual-band lidar method combined with multidimensional statistical moments provides additional parameters, improving species identification of aero-fauna. By systematically applying dimensionality reduction based on multidimensional moments, the dataset from a tropical virgin forest was reduced to a compact set of parameters that reflect instrument characteristics, flight heading and species-specific feature combinations such as the size ratio of the body and wings. Observed signal asymmetries further allowed assessment of coarse insect flight headings in two dimensions (ascending or descending flight and flying away from or towards the receiver) beyond previously described transverse velocities. Group-specific cross-section ratios between 808 nm and 980 nm signals enable coarse insect classification without time and labor-intensive machine learning procedures. We want to emphasize that compressing data from 2D to 0D carries the risk of losing information, such as periodic time–frequency features, during the reduction from 1D to 0D.

The range resolution could be improved by combining time-of-flight techniques with Scheimpflug lidar (Wang et al., 2025), by implementing frequency combs in lidar systems (Kuse and Fermann, 2019), or by estimating axial velocities using heterodyne Doppler lidar methods (Wainwright et al., 2017).

The accuracy of cross-section estimates could be enhanced, for example, by determining the observation angle from higher harmonics in the signal (Gebru et al., 2018; Li et al., 2023b). Furthermore, it may be possible to distinguish whether transits occur peripherally or through the center of the pixel footprint by analyzing the kurtosis of the temporal envelope: transits through the pixel footprint are expected to exhibit a top-hat temporal envelope with sharp edges and low temporal kurtosis.

Similarly, improvements in the accuracy of apparent size estimates could be achieved by evaluating the degree of focus and the kurtosis of the recorded echoes (see *p*^4^_1_, Optical focus, in Table 1).

Comparisons of signal strength between wavelengths using mean cross-section (σ_mean_, σ_1_^0^), spread (σ_spread_, σ_2_^0^), skewness (*p*_skew_, *p*_1_^3^), and kurtosis (*p*_kurt_, *p*_1_^4^) enhance the quality of cluster separation (Fig. S5). Strategic positioning of the lidar system, for example at insect migration corridors or between foraging sites and beehives, could theoretically enable monitoring of flight trajectories. General trends in reflectivity related to insect melanization were observed, along with specific cases indicating exceptions associated with particular taxa. These results demonstrate that dual-band entomological lidar signals contain more detailed taxonomic and behavioral information than single wavelength bands. Thus, multidimensional parametrization combined with additional wavelength bands offers expanded capabilities for insect discrimination.

## Supplementary Material

10.1242/jexbio.251761_sup1 Supplementary information
