# Supplementary material for: Robust and diverse multidimensional statistical moments in dual-band entomological lidar for improved real-time insect monitoring
Source: J Exp Biol. 2026 May 28;229(11):jeb251761. doi: 10.1242/jeb.251761 (PMC13286368; doi:10.1242/jeb.251761)
Supplement: Supplementary information [file jexbio-229-251761-s1.pdf]

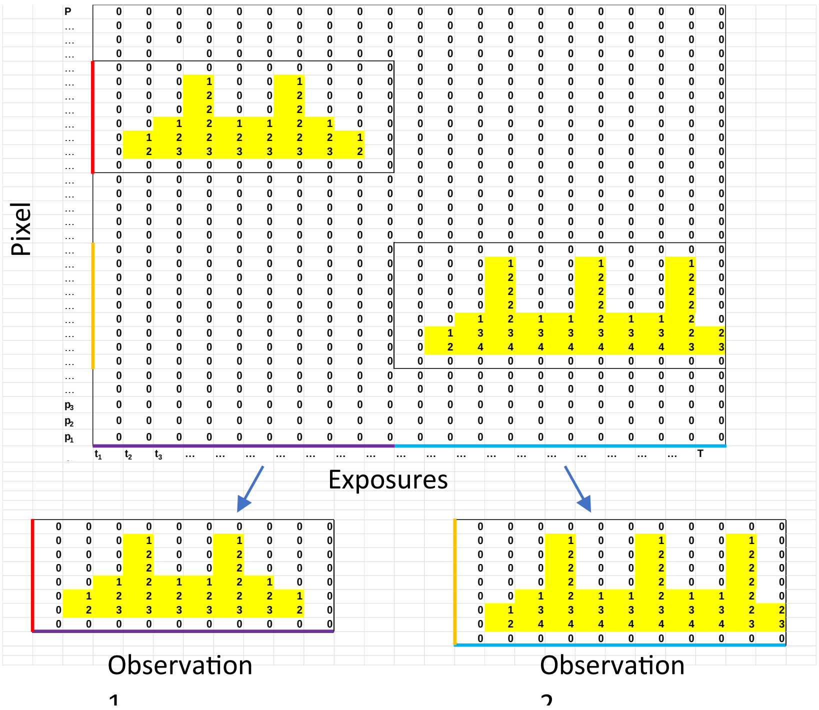

Fig. S1. Simplified scheme explaining the basic cropping procedure. A snippet of raw data is processed using Boolean masks (threshold in this example >0) to determine the boundaries around the backscatter (Observation 1 & 2) encoding areas.

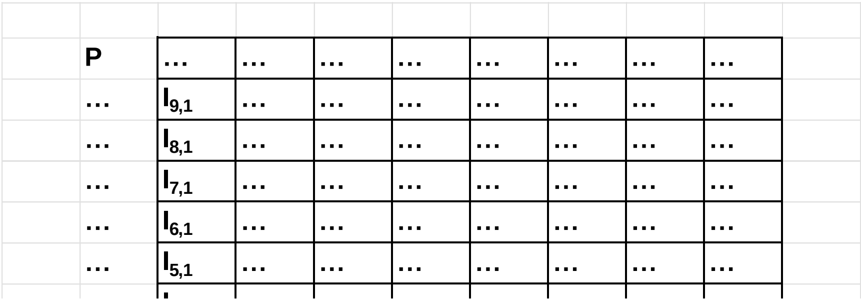

Fig. S2. Scheme explaining the basic data format. So let Ip,t be a P by T matrix, where P is the number of pixels (range), and T is the number of exposures (time), Ip,t represents the signal strength of pixel p during exposure t.

Journal of Experimental Biology • Supplementary information

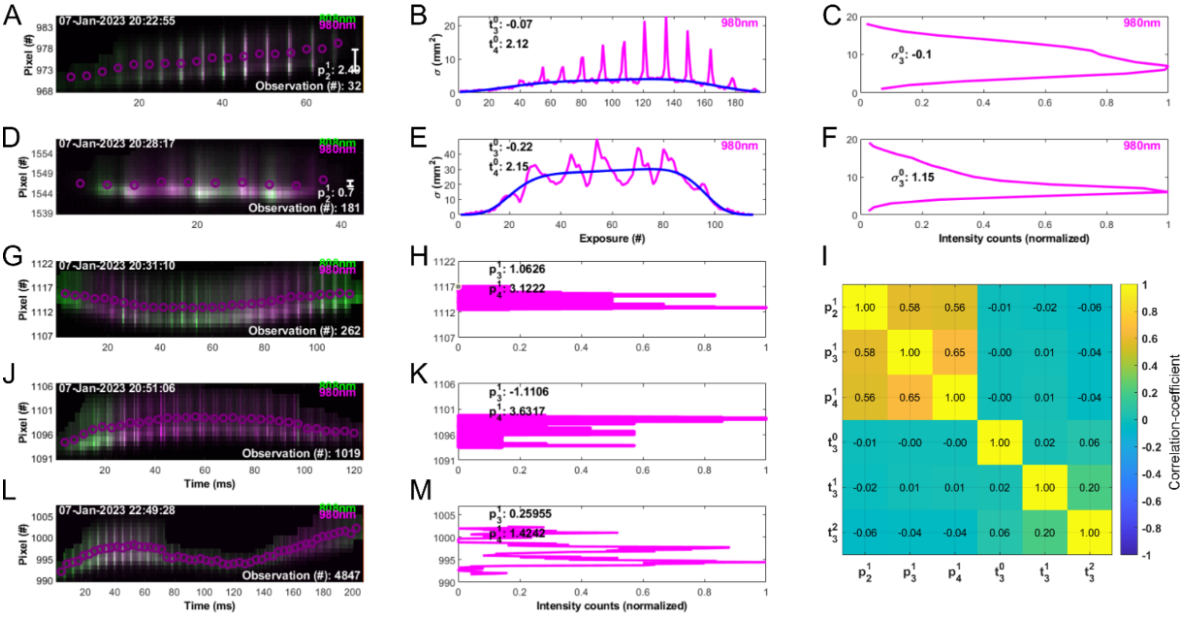

Fig. S3: Explanation of some of the Statistical moments mentioned in Table 1. A, D: Time range maps showing the $p_{CoM(t)}$s of the 980nm signal (only every 20th datapoint plotted) of 2 insect observations as purple circles. The white bars mark the spread ($\mathbf{p}_2^1$) of the $p_{CoM(t)}$s, which can be used to approximate the displacement of the insects' bodies while crossing the Lidar beam. To provide a better overview, only $p_{CoM(t)}$s of the 980nm signal is shown. B, E: Cross-sections corresponding to the observations in A and D. The blue curve was fitted in both curves to illustrate the skewness ($\mathbf{t}_3^0$) and kurtosis ($\mathbf{t}_4^0$) of both cross-sections. A negative skewness as shown in B indicates an increase in signal strength at the end of the observation, which in turn indicates that the insect was following a trajectory towards the receiver. The kurtosis can give rise to the overall envelope shape of the insect observation, which can be of service for clustering purposes. C, F: Histogramms of the cross-sections in B and E in the pixel-domain to depict the skewness ($\boldsymbol{\sigma}_3^0$) of the respective curves. A negative skewness as in C indicates a more specular rather than a more diffuse reflection of the wings which is the case in F. G, J, L: Time range maps showing the $p_{CoM(t)}$s of the 980nm signal (only every 20th datapoint plotted) of 3 insect observations as purple circles (like in A and D). Note that the respective insects apparently followed a curved trajectory as they passed through the laser beam which was bend downwards (G), upwards (J) and both, up- and down-wards (L). The corresponding histograms of the $p_{CoM(t)}$s in (H, K, M) were plotted to illustrate the skewness ($\mathbf{p}_3^1$) and the kurtosis ($\mathbf{p}_4^1$) of the $p_{CoM(t)}$s in the pixel domain. A positive (H) or negative (K) skewness can indicate that the insect was following an upwards (J) or downwards (G) trajectory, while e.g. a low kurtosis value (M) indicates that the insect changed its' course multiple times while it was crossing the beam (L). To provide a better overview, only the $p_{CoM(t)}$s of the 980nm signal is shown. I: Cross-correlogram depicting the correlation of the trajectory-related statistical moments (moments shown in blue in Table 1), indicated by the color-coded correlation coefficient.

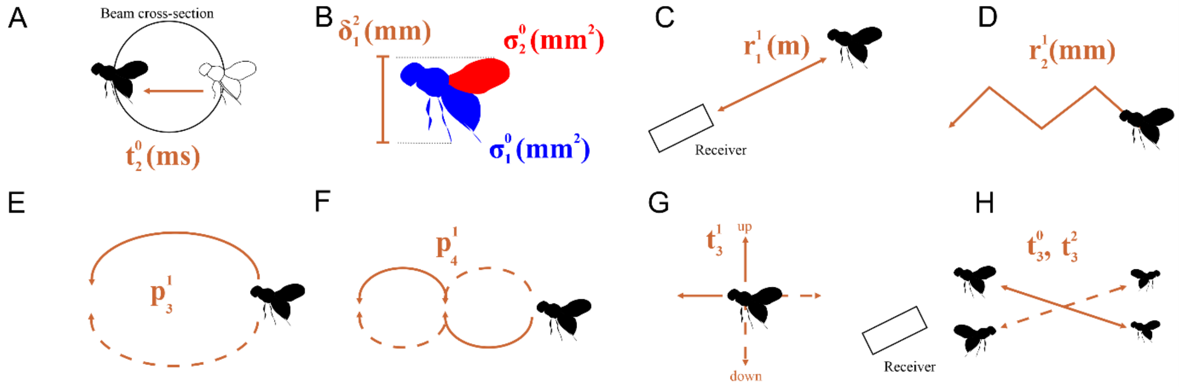

Fig. S4. A-H: A series of schemes where a specific statistical moment is depicted using a simplistic illustration. A, transit time; B, size and cross-section of body and wing; C, distance/altitude; D, displacement; E, curvilinearity; F, erraticness; G, altitude increase/ decrease. H, signal/angular size increase or decrease.

Journal of Experimental Biology • Supplementary information

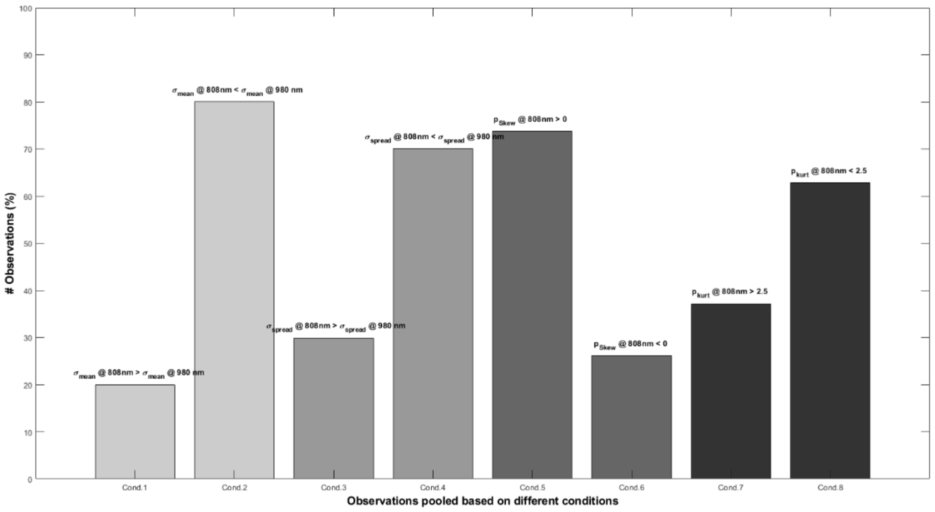

Fig. S5. The bar plot shows the dataset of observations filtered based on different conditions. The respective condition (Cond.1-8) is plotted on top of the individual bars, which encode the percentage of observations falling into the respective condition. Conditions 1, 2, 3 and 4: A comparison of the 808nm and 980nm band signal strengths. The bars indicate how many observations showed a bigger or smaller body (σ_{mean}) or wing (σ_{spread}) cross-section in the 808nm or the 980nm band respectively. Conditions 5 and 6: The bars encode the percentage of positive and negative skewness of the signals distribution over the respective range of pixels (p_{skew}). Data for 980nm not shown. Conditions 7 and 8: The bars encode the percentage of kurtosis values above or under the value 2.5 of the signals distribution over the respective range of pixels (p_{kurt}). Data for 980nm not shown. The value 2.5 was chosen since the data seem to cluster around 2.5 (compare Fig.S8, G).

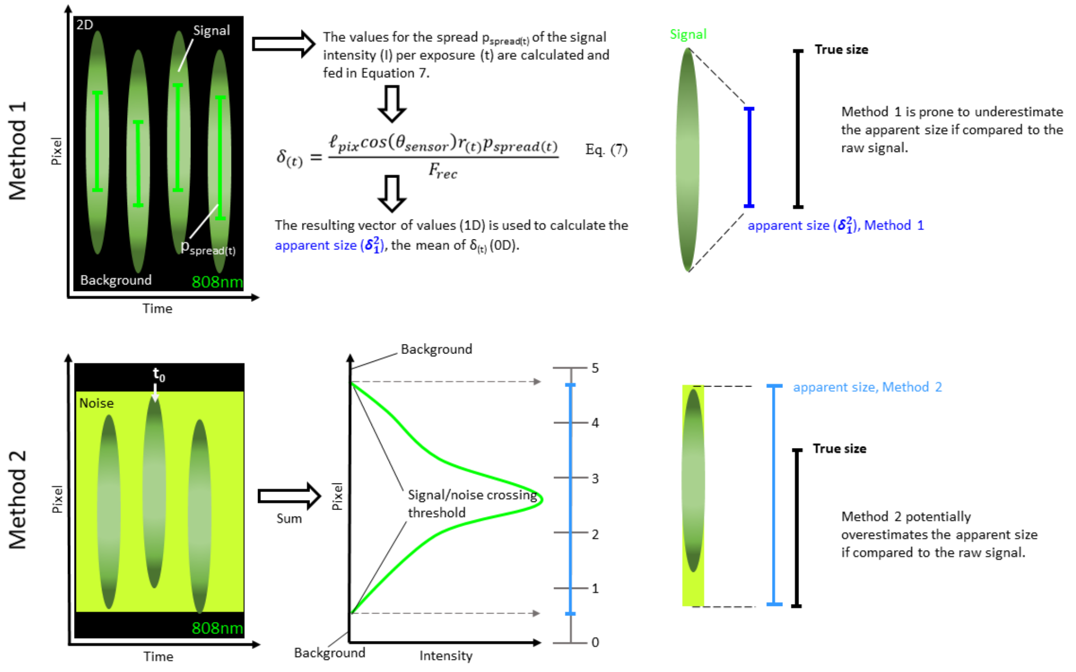

Fig. S6. A scheme depicting the simplified concepts of calculating the apparent size using the two suggested methods. Method 1 uses the spread of the signal intensity over the respective pixels across all exposures. Method 2 thresholds only the pixels at t_0 and projects the resulting size on a previously approximated scale. Here, the problem might be that noise masks the signal size due to cropping artefacts.

Journal of Experimental Biology • Supplementary information

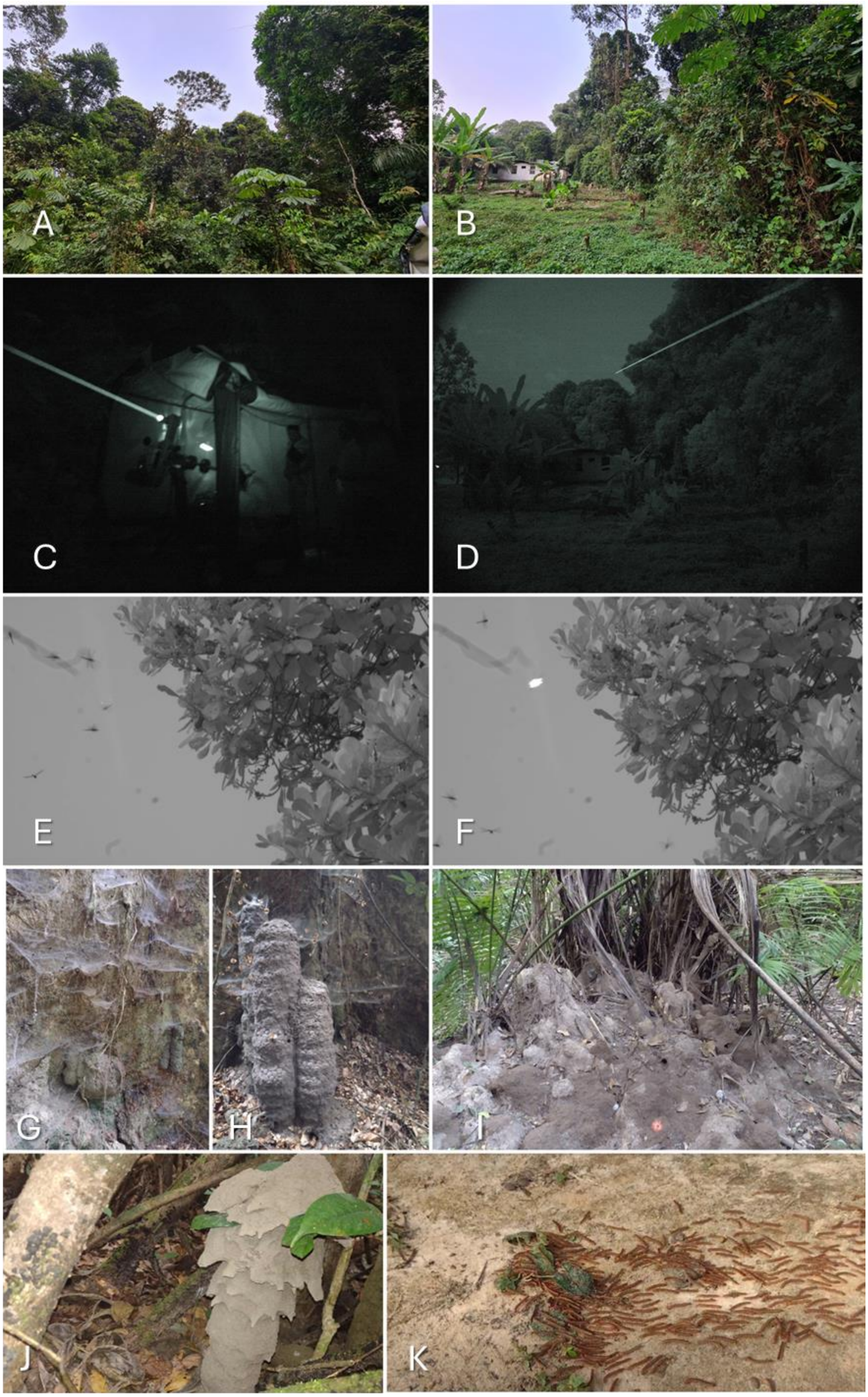

Fig. S7. Overview of the study site and experimental configuration. (A–B) Characterization of the forest edge where the LiDAR system was deployed, showing associated vegetation including banana trees and other vegetation. (C–D) Infrared imagery of the LiDAR apparatus and beam trajectory probing along the forest edge and above the canopy. (E–F) Dragonfly (Odonata) swarming activity recorded right after sunset; (F) captures a specific instance of a dragonfly intersecting the beam, resulting in a visible backscatter flash. (G–K) Surrounding ecological context, including arthropod presence.

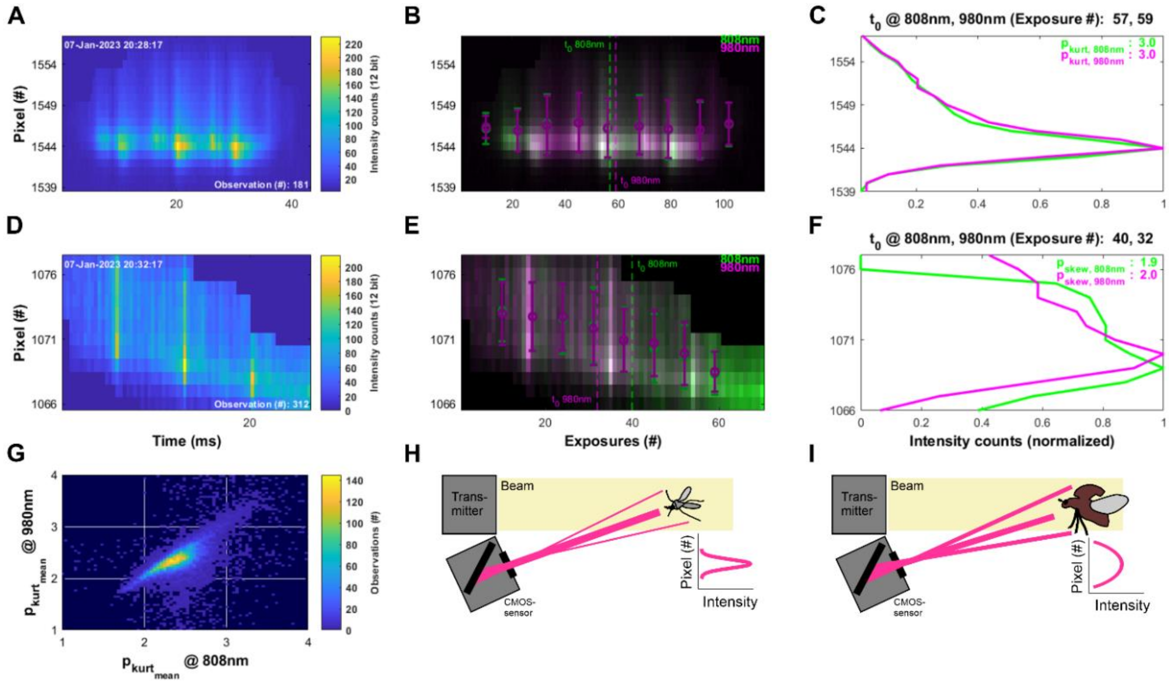

Fig. S8. A comparison of signal kurtosis in the 808 nm and 980 nm bands. (A, D) Time-range maps of two insect recordings. (B, E) Dual-band time-range maps in false colors (green = 808 nm, magenta = 980 nm). Circles show the center of mass of the intensity distribution; vertical error bars depict the spread. (C, F) Normalized intensity distributions from (B, E). (G) 2D histogram comparing kurtosis in the 808 nm vs. 980 nm bands. (H, I) Schematic illustrating how signal kurtosis relates to overall insect reflectivity (e.g., thorax/abdomen size, melanization).

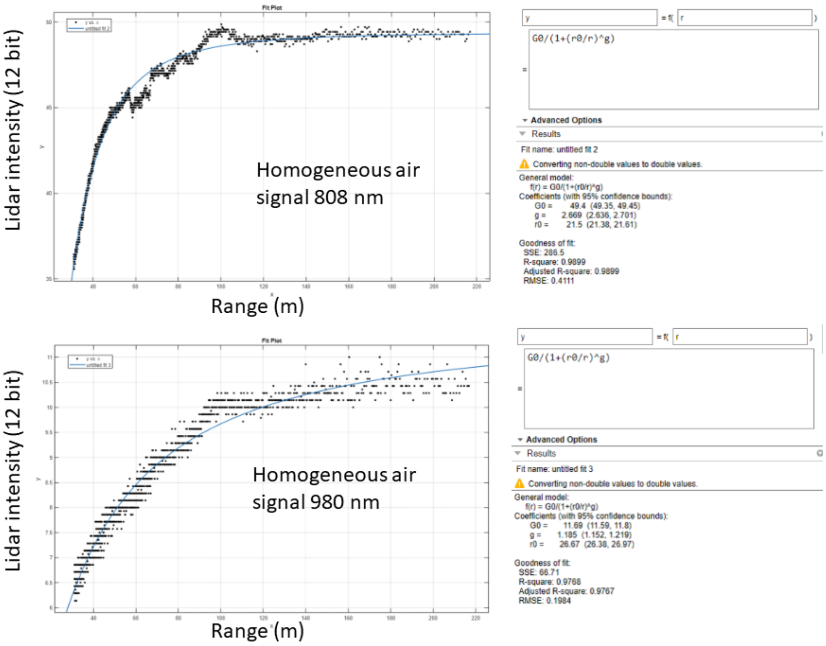

Fig. S9. A range sensitivity curve is estimated by fitting an analytical curve the static air signal with the assumption that the monitored air at the given time is homogeneous. The model displays adjusted R^2 correlation with data of 98.88% and 97.67% for the 808 nm and 980 nm band respectively. The parameter G0 in the fit is later rescaled by directing the beam at a black neoprene target, Eq.8. (Fig.9s display a case where the beam is directed at the sky). The 95% confidence intervals for the g (γ) factor is ± 2% for 808 nm band and ± 3% for the 980 nm band, the $r_{½}$ (r0 in formula above) factor is ± 0.5% for the 808 nm band and ± 1% for the 980 nm band.
